# Continuous autotrophic production of alcohols with *Clostridium autoethanogenum* LAbrini in a submerged membrane bioreactor

**DOI:** 10.1186/s40643-026-01128-y

**Published:** 2026-09-26

**Authors:** Anne Oppelt, Nadja Sommer, Anna Stock, Daniel Bischoff, Dirk Weuster-Botz

**Affiliations:** School of Engineering and Design, Chair of Biochemical Engineering, Technical University of Munich, Boltzmannstr. 15, 85748 Garching, Germany

**Keywords:** Syngas fermentation, Membrane bioreactor, Continuous process, Submerged microfiltration membrane, Cell retention, *Clostridium autoethanogenum* LAbrini, Carbon monoxide, Ethanol, D-2,3-butanediol.

## Abstract

Due to the generally low growth rates of acetogens, conventional batch and continuously operated syngas fermentation processes in suspension cultures, e.g., stirred tank bioreactors, typically result in low cell concentrations and, thus, limited volumetric productivities (space-time yields). To increase cell concentrations in syngas fermentations, a continuously operated submerged microfiltration membrane bioreactor (MBR) was applied on a L-scale with *C. autoethanogenum* LAbrini for the production of alcohols. Cell retention was achieved by inserting a sintered metal microfiltration rod into a standard stirred-tank bioreactor. A batch phase followed by continuous operation with full cell retention until 95% CO conversion was used to start the MBR operation with partial cell retention. Three continuous syngas fermentation processes were operated until a quasi-steady state was achieved with total process times of up to 10 days. Higher cell (4.4 g L^−1^), ethanol (4.8 g L^−1^), and D-2,3-butandiol (0.9 g L^−1^) concentrations, and very much improved volumetric productivities were achieved at a medium dilution rate (1.4 d^−1^) below the maximum growth rate of *C. autoethanogenum* LAbrini, compared to an autotrophic batch process used as reference. However, at a medium dilution rate (2.8 d^−1^) above the maximum growth rate, solely the volumetric productivities were improved, e.g., 7.73 g L^−1^ d^−1^ ethanol compared to 0.44 g L^−1^ d^−1^ in the reference batch process. The start-up time of the MBR operation with partial cell retention was successfully reduced by applying an initial mixotrophic batch phase with fructose as a heterotrophic carbon source. The submerged microfiltration membrane rod made of sintered metal, applied here for the first time in a stirred-tank bioreactor for cell retention, demonstrated long-term operational stability in continuous gas fermentations with cell concentrations up to 4.4 g L^−1^. Due to its low specific membrane area of ~ 70 cm^2^ L^− 1^, the scalability of a submerged MBR is facilitated, especially when operated at the relatively low permeate flow rates of < 1.65 mL cm^−2^ h^−1^, as demonstrated here.

**Graphical abstract:**

**Figure:**
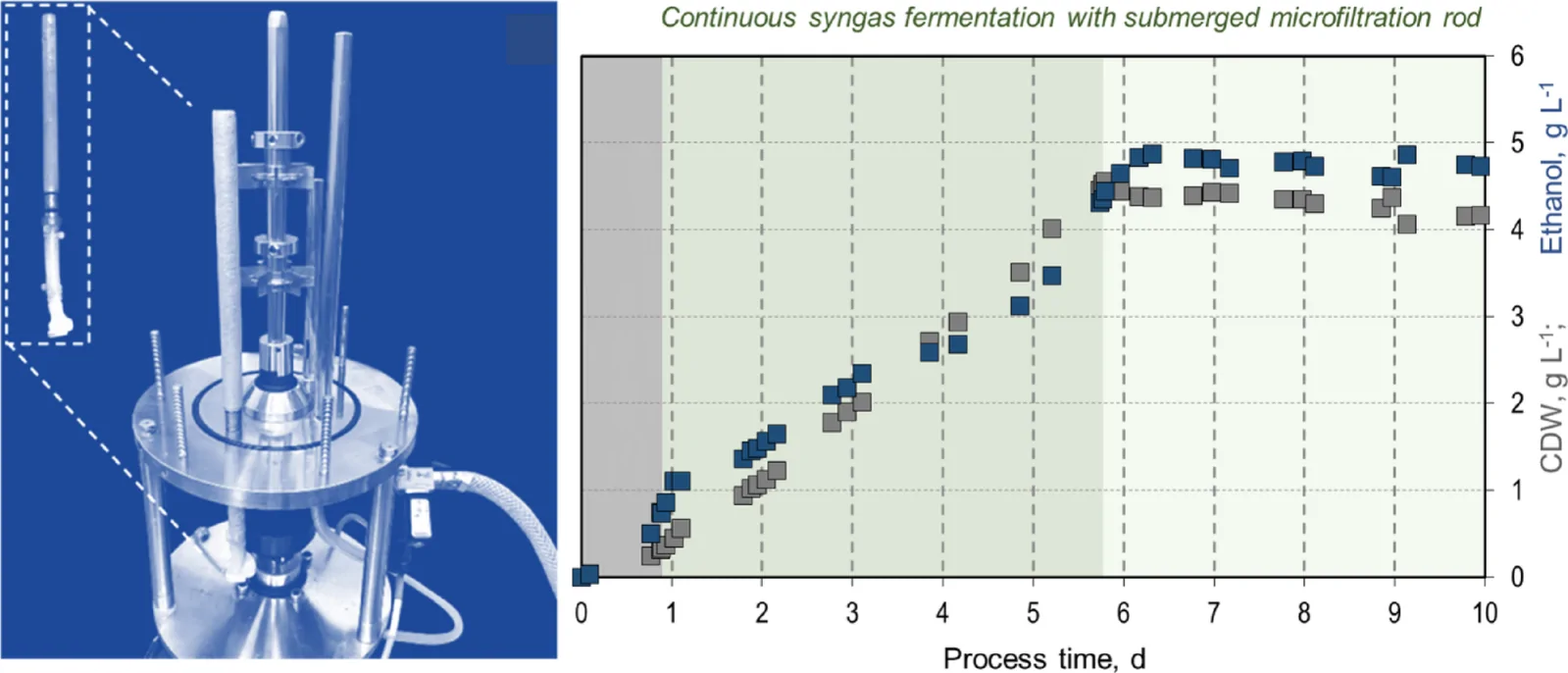

**Supplementary Information:**

The online version contains supplementary material available at https://doi.org/10.1186/s40643-026-01128-y.

## Introduction

Syngas fermentation is part of a hybrid thermochemical-biotechnological conversion process for the production of biofuels and platform chemicals (Phillips et al. 2017, Sun et al. 2019). Syngas is generated by thermochemical gasification of renewable feedstocks or obtained from industrial waste gas (Benevenuti et al. 2021, Devarapalli et al. 2017). The main components of syngas, carbon monoxide (CO), carbon dioxide (CO_2_), and hydrogen (H_2_) gases, can be metabolized after solving in aqueous media by anaerobic acetogenic microorganisms during syngas fermentation (Benevenuti et al. 2021, Phillips et al. 2017, Poehlein et al. 2025). Alternatively, syngas can be converted into hydrocarbons via the Fischer-Tropsch process using metal catalysts at high temperatures and pressures (Rofer-DePoorter 1981). In contrast to these chemical catalysts, acetogenic microorganisms demonstrated superior tolerances to various syngas impurities and enable operation under milder conditions, such as lower temperatures and lower pressures, while reducing catalyst costs (Abubackar et al. 2011, Daniell et al. 2012, Munasinghe and Khanal 2010). However, the successful industrial application of syngas fermentation strongly relies on the microorganism used, the product formed, the type and configuration of the bioreactor, and the syngas composition (Asimakopoulos et al. 2018, Daniell et al. 2012, Owoade et al. 2023).

Although all acetogens use the reductive acetyl-CoA pathway, also known as the Wood-Ljungdahl pathway (WLP), the formation of the final products depends on the species employed (Bengelsdorf et al. 2013, Mock et al. 2015, Poehlein et al. 2025). Different acetogenic species produce distinct product spectra, ranging from acetate as the sole product to mixtures of acids and alcohols (Poehlein et al. 2025). The closely related species *C. autoethanogenum*, *C. ljungdahlii*, and *C. ragsdalei* exclusively produce acetate, ethanol, and 2,3-butanediol (Poehlein et al. 2025).

The WLP converts CO, CO_2_, and/or H_2_ into the key metabolic intermediate acetyl-CoA, which serves as a precursor for both anabolism and catabolism. CO and H_2_ can act as electron donors, with CO being the thermodynamically preferred electron source (Drake et al. 2008, Mock et al. 2015).

A significant bottleneck in syngas fermentation is the poor solubility of CO and H_2_ in aqueous media (Weuster-Botz 2021). Due to the low water solubility, continuous dispersion of the gas phase is required in the aqueous fermentation medium to ensure sufficient substrate availability for microbial conversion. Thus, the gas-liquid mass transfer rate is an important process variable, strongly affected by the (local) partial pressures of these gas components, the power input, and the gassing rate (Maru et al. 2018, Munasinghe and Khanal 2010, Weuster-Botz 2021). Therefore, it is not surprising that increasing reactor pressure can enhance acetogen productivity (Hecke et al. 2019). This is inherent in industrial syngas fermentations due to the hydrostatic pressures caused by liquid columns up to 30 m (Poehlein et al. 2025). However, elevated CO concentrations can have inhibitory effects (Hurst and Lewis 2010, Mayer et al. 2018).

In addition to assimilating C1 molecules via the WLP, acetogens also possess heterotrophic metabolic pathways for the conversion of hexoses and pentoses (Fast et al. 2015). For instance, *C. scatologenes* can metabolize a wide variety of hexoses and pentoses, including glucose, sucrose, galactose, xylose, and glycerol (Maru et al. 2018). In contrast, *C. autoethanogenum* and *C. ljungdahlii* are limited to fructose, xylose, and arabinose (Maru et al. 2018). Furthermore, several studies have demonstrated that acetogens can simultaneously metabolize C1 gases and C5/C6 carbohydrates, enabling mixotrophic growth (Abubackar et al. 2016, Fast et al. 2015, Hill et al. 2025, Jones et al. 2016, Mann et al. 2020, Schwarz et al. 2024). As shown with *C. autoethanogenum* JA1-1 and its mutant *C. autoethanogenum* LAbrini, organic carbon supplementation (xylose and arabinose) significantly enhances the production of the favoured alcohols (ethanol and 2,3-butanediol), and improves the alcohol-to-acetate ratio in batch processes in stirred tank bioreactors. Further, mixotrophic growth with xylose or arabinose also markedly improved CO utilization (Oppelt et al. 2024, Oppelt et al. 2026). As a consequence, acetogenic mixotrophy offers a promising route to overcome the limitations of purely autotrophic syngas fermentation by improving growth and CO conversion and redirecting carbon flux toward more reduced products (alcohols).

The generally low growth rates of acetogenes result in low volumetric productivity (space-time yield) in autotrophic batch processes. To achieve higher volumetric productivities, the most common strategy is to use continuously operated bioreactors (Weuster-Botz 2021). For example, at pilot and industrial scales, continuously operated bubble columns or gas-lift reactors are the standard for syngas fermentations with acetogens (Daniell et al. 2012, Elisiário et al. 2022, Poehlein et al. 2025, Weuster-Botz 2021). At laboratory scale, CSTRs are frequently used and typically operated as chemostats (Abubackar et al. 2016, Asimakopoulos et al. 2018, Doll et al. 2018, Munasinghe and Khanal 2010). However, in chemostats, the dilution rate is limited by the maximum specific growth rate of the microorganism used. Hence, decoupling biomass residence time from the mean hydraulic residence time of the aqueous phase prevents cell washout and enables operations at dilution rates exceeding the maximum specific growth rate, resulting in higher volumetric productivities (Asimakopoulos et al. 2018). Typical bioreactor configurations are membrane bioreactors (MBRs) with microfiltration membranes or biofilm reactors, such as trickle-bed biofilm reactors (Asimakopoulos et al. 2018, Elisiário et al. 2022, Kantzow et al. 2015, Mayer et al. 2018, Munasinghe and Khanal 2010). For MBRs, cell retention can be realized using submerged membranes in a stirred-tank bioreactor or external microfiltration membranes operated in a bypass (Munasinghe and Khanal 2011). However, bypass-based cell retention may be suboptimal for syngas fermentation. The low solubility of gaseous substrates in an aqueous solution means that dissolved gases can be rapidly depleted at high cell densities. During recirculation through a bypass, cells may therefore experience severe substrate limitation, which could potentially trigger physiological stress responses and even partial cell inactivation. By contrast, MBRs with submerged microfiltration membranes do not suffer from substrate limitation, unlike an external bypass. However, the major drawback of MBRs with submerged microfiltration membranes is that the membranes can not be replaced or cleaned during operation, which may result in membrane fouling and thus reduced filtrate flow rates over time (Carstensen et al. 2012).

Increased volumetric productivities have already been demonstrated, for instance, with an MBR equipped with submerged microfiltration hollow fibers in a lab-scale stirred-tank bioreactor used for gas fermentation with *Acetobacterium woodii*. A volumetric acetate productivity (space-time yield) STY_acetate_ of 147.8 g L^−1^ d^−1^ was achieved at a dilution rate of 8.4 d^−1^. In contrast, a CSTR reached only a STY_acetate_ of 18.2 g L^−1^ d^−1^ at a dilution rate of 0.84 d^−1^ (Kantzow et al. 2015). MBRs with submerged membranes are already being established in full-scale wastewater treatment plants (Al-Asheh et al. 2021, Xiao et al. 2019).

*C. autoethanogenum* is considered one of the most promising acetogens due to its successful industrial implementation by LanzaTech (Liew et al. 2022, Poehlein et al. 2025). Since 2024, the novel derivate *C. autoethanogenum* LAbrini has been commercially available, having been engineered through adaptive laboratory evolution (ALE) and reverse genetic engineering by Ingelmann et al. (Ingelman et al. 2024). *C. autoethanogenum* LAbrini was shown to exhibit faster growth, reduced acetate formation, and greater robustness in CSTRs compared to its wild-type strain *C. autoethanogenum* JA1-1 (Ingelman et al. 2024, Ingelman et al. 2025). Additionally, *C. autoethanogenum* LAbrini demonstrated improved performance in mixotrophic batch processes compared to the wild-type strain (Oppelt et al. 2026). However, all published gas fermentation studies with *C. autoethanogenum* LAbrini remain limited to batch and chemostat operations in stirred-tank bioreactors under atmospheric pressure.

In this work, we operated a lab-scale CSTR as a continuous gassed MBR with a novel submerged microfiltration membrane in syngas fermentations to decouple biomass (*C. autoethanogenum* LAbrini) from mean hydraulic residence times. The MBR is operated autotrophically with continuous gassing at dilution rates even above the maximum specific growth rate of *C. autoethanogenum* LAbrini enabling higher volumetric productivities (space-time yields) than in autotrophic batch processes. Efficient start-up of the MBR was achieved with a first autotrophic or mixotrophic batch phase, followed by continuous autotrophic MBR operation with total cell retention to enable rapid biomass accumulation. To avoid gas-liquid mass transfer limitations at increased biomass concentrations in the MBR, a continuous bleed was initiated, withdrawing a portion of the cell suspension once CO conversion exceeded 95%. This ensures the establishment of defined autotrophic steady-states in the MBR operated with continuous gassing.

## Materials and methods

### Microorganism, medium, and pre-cultures

*C. autoethanogenum* LAbrini (DSM 115981) was purchased from the German Collection of Microorganisms and Cell Cultures (DSMZ, Braunschweig, Germany) as an active culture. The procedure described in our previous study was followed to prepare the master cell bank (MCB) and the working cell bank (WCB) (Oppelt et al. 2026). MCB and WCB were stored in sterile, anaerobic Hungate tubes (16 × 125 nm, Dunn Labortechnik, Asbach, Germany) at − 80 °C.

The medium for pre-cultures, the initial batch phase of the membrane bioreactor (MBR), and the feed medium were prepared according to Doll et al. (Doll et al. 2018). Its detailed composition is listed in Supplementary Information (Table S1).

15 g L^−1^ 2-(N-morpholino)ethanesulfonic acid (MES) was added to the medium of the pre-cultures as a buffer. The medium was boiled for 20 min on a heating mantle and then purged with N_2_ for at least 30 min to establish anaerobic conditions. 100 mL of the medium was transferred into 250 mL or 500 mL anaerobic bottles under an anaerobic atmosphere. The anaerobic bottles were then autoclaved (Systec VX-150, Systec GmbH & Co. KG, Linden, Germany).

For the process with the autotrophic batch phase, an autotrophic pre-culture was prepared, while for the processes with mixotrophic batch phases, mixotrophic pre-cultures were used. Detailed preparation of pre-cultures is described in our previous study (Oppelt et al. 2026).

A 5 L bottle (Duran borosilicate glass, GL45, Schott AG, Mainz, Germany) containing 4 L of medium was sterilized by autoclaving (Systec VX-150, Systec GmbH & Co. KG, Linden, Germany). After sterilization, anaerobization was initiated after cooling the medium to 80 °C and gassing with a mixture of N_2_ and CO_2_ (80:20) at a flow rate of 25 NL h^−1^ via sterile filters. Sterile 10 mL L^−1^ vitamin solution and 0.5 mL L^−1^ polypropylene glycol 400 (PPG400) were added aseptically via a syringe and septum after the temperature was below 40 °C. After 4 h, 0.4 mL L^−1^ of anaerobic L-cystein hydrochloride was added aseptically as a reducing agent, and anaerobization was stopped. The anaerobic medium was then stored at 4 °C until further use.

Cells from the second pre-culture stage at the end of the exponential growth phase were harvested anaerobically for inoculation (10 min, 3620 rcf, Rotica 50 RS, Hettich GmbH & Co. KG, Tuttlingen, Germany). All MBR processes were inoculated with cells resuspended in 10 mL of anaerobic phosphate-buffered saline solution (12 mM phosphate). The initial cell dry weight (CDW) concentrations in the MBR were 0.03 g L^−1^.

### MBR operation

**Fig. 1:**
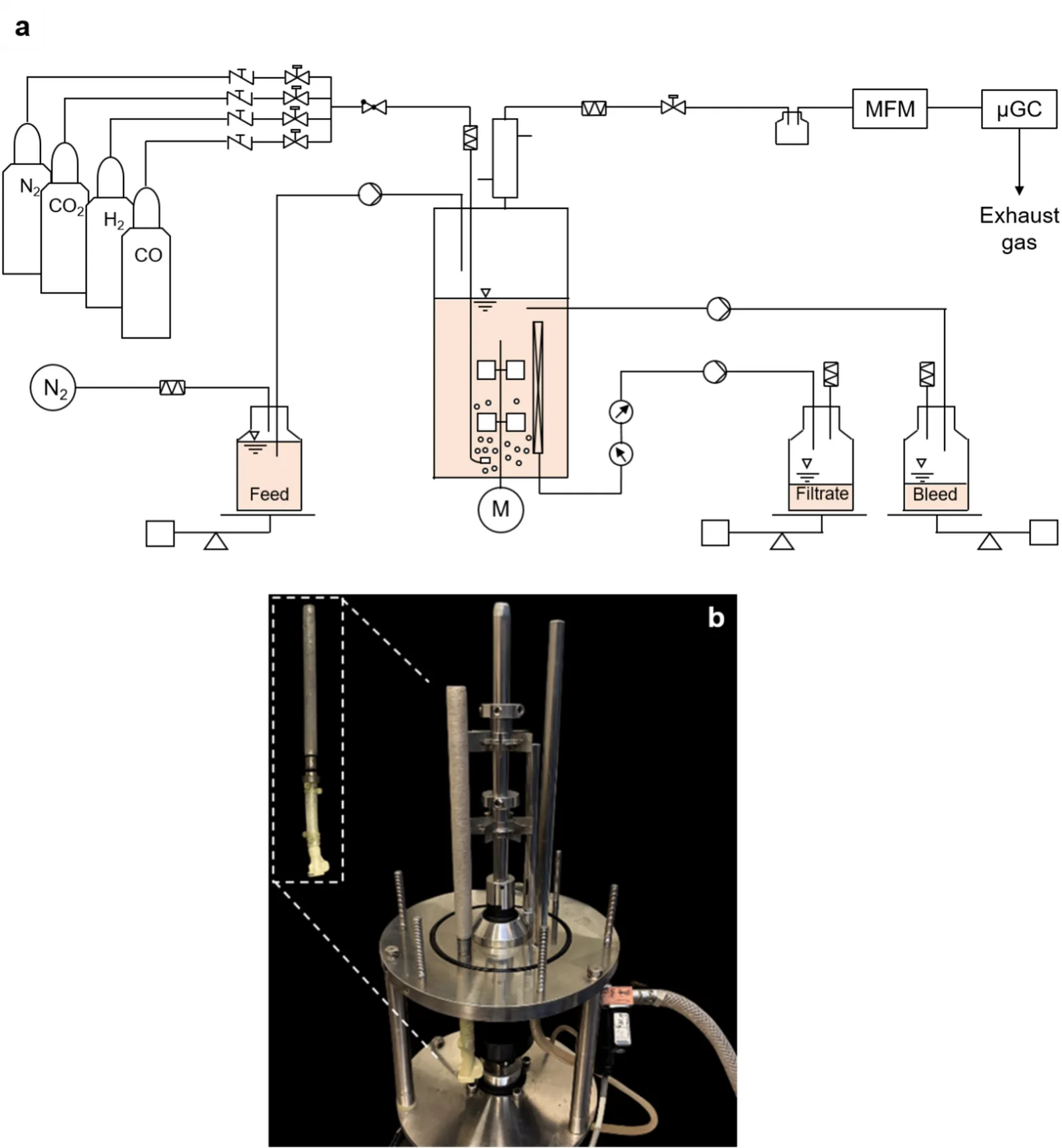
**a** Scheme of the set-up for continuous gas fermentation in a standard stirred-tank bioreactor with a submerged sintered microfiltration membrane rod. **b** Photo of the sintered microfiltration membrane rod fixed at the bottom of the stirred tank bioreactor beneath the stirrer axis with the two Rushton turbines. The cylindrical bioreactor housing, made of steel, and the baffles are not installed for visualization

The MBR processes were carried out in a fully controlled 2.4-L stainless steel stirred-tank bioreactor (STR) with baffles (KLF2000, Bioengineering, Wald, Switzerland) with a working volume $\:{\mathrm{V}}_{\mathrm{Reactor}}$ of 1 L (Fig. 1a,b). The STR with an inner diameter of 98 mm was agitated at 1200 rpm (15.1 W L^−1^) using two six-bladed Rushton turbines. A sterilizable probe was used for pH monitoring (pH: 405-DPAS-SC-K8s/120). 3 M NaOH or 0.5 M H_2_SO_4_ was used to control pH 6.0 in the bioreactor. Temperature was maintained at 37 °C using a sensor, a heating rod, and a cooling rod. Pressure was controlled at 2.5 bar_abs_. A pressure probe and a mechanical safety valve that opens at 3.5 bar_abs_ were installed for safety purposes.

Syngas was supplied via the lid through a tube attached to the baffles, equipped with a sintered frit at the end, which was placed below the first Rushton turbine near the bottom of the STR. Exhaust gas was cooled to 2 °C after passing through the lid to minimize evaporation. The bioreactor was continuously gassed with an artificial syngas mixture with a total flow rate of 5 NL h^−1^ (0.083 vvm). Artificial syngas composed of 1.95 NL h^−1^ N_2_ (39.4 (v/v)), 1.50 NL h^−1^ CO (29.8 (v/v)), 1.10 NL h^−1^ H_2_ (22.0 (v/v)), and 0.45 NL h^−1^ CO_2_ (8.9 (v/v)) was used (Oppelt et al. 2024, Oppelt et al. 2026, Rückel et al. 2022).

The feed medium was supplied through a lid port, while the submerged sintered microfiltration membrane rod was fixed at a standard port at the reactor’s bottom. The unfiltered fermentation broth (bleed) was withdrawn at a side port. The MBR with the submerged microfiltration membrane rod containing 1 L medium (without vitamins, L-cysteine hydrochloride, D-fructose, and MES) was sterilized in situ. After cooling to 37 °C, 10 mL of the vitamin stock solution was added aseptically. MBR and medium were anaerobized with sterile 20 NL h^−1^ N_2_ for at least 2 h. At least 16 h before inoculation, the artificial syngas composition was adjusted with 5 NL h^− 1^. Immediately prior to inoculation, 0.4 g L^−1^ sterile, anaerobic L-cysteine hydrochloride was added. Additionally, if the MBR was operated with a mixotrophic batch phase, sterile, anaerobic D-fructose was added until approximately 18 g L^−1^ was achieved in the reactor medium. Immediately after inoculation, a pressure of 2.5 bar_abs_ was established via a pneumatic control valve.

**Table 1:** Initial D-fructose concentrations in the batch phases, and dilution rates of feed, filtrate, and bleed of the three continuous autotrophic MBR processes with *C. autoethanogenum* LAbrini

MBR	Batch phase	Continuous phase I Total cell retention		Continuous phase II Partial cell retention		
	c_initial, D−Fructose_, g L^−1^	D_Feed_, d^−1^	D_Filtrate_, d^−1^	D_Feed_, d^−1^	D_Filtrate_, d^−1^	D_Bleed_, d^−1^
(I)	0	1.4	1.4	1.4	1.0	0.4
(II)	17.8	1.4	1.4	1.4	1.0	0.4
(III)	18.3	2.8	2.8	2.8	2.0	0.8

Table 1 shows the dilution rates during total and partial cell retention of three continuous syngas fermentation processes in the MBR. Following a 22 h initial batch phase, the MBR was operated first with total cell retention to enable fast accumulation of the *C. autoethanogenum* LAbrini cells. To prevent CO limitation, the bleed was started as soon as CO conversion in the gas phase reached 95%. The dilution rate of the bleed ($\:{\mathrm{D}}_{\mathrm{Bleed}}$) was adjusted to the specific growth rate measured in the MBR during total cell retention ($D_{{{\mathrm{Bleed}}}} = \mu _{{{\mathrm{total}}\;{\mathrm{cell}}\;{\mathrm{retention}}}}$) to keep the dry cell mass concentration constant.

### Submerged sintered microfiltration membrane rod

A custom-made sintered microfiltration membrane rod (GKN Sinter Metals Filters, Rodevormwald, Germany) with an overall length of 179 mm was equipped with an external thread (M8 × 1.5) for installation at a standard port at the bottom of the STR. The cylindrical asymmetrical microfiltration membrane rod with a length of 157 mm, an inner diameter of 10 mm, and an outer diameter of 14 mm was made of a stainless steel support coated on the outer surface with a porous metal mixture (Fe, Cr, Ni, C, Mo, Si) manufactured by an isostatic compacting process and features an outer membrane surface area of 70.6 cm^2^ with mean pore sizes of 0.1 μm (pore size distribution and porosity are unknown). The hydrophilic membrane is designed to withstand a transmembrane pressure difference of up to 130 bar and was operated at permeate fluxes ranging from 5.9 to 16.5 L m^−2^ h^−1^. (41.65–116.49 mL h^−1^).

As shown in Fig. 1b, the membrane was screwed to a bottom port of the reactor using a double nipple and a sealing ring. A pipe section welded to the double nipple protrudes from the outer/lower side of the reactor’s bottom. On the pipe section, a coupling hose connection with a shut-off valve (CPC, Colder Products Company, St. Paul, USA) was fixed to couple the filtrate connection before initiating the continuous operation of the MBR. Transmembrane pressure between the MBR and the membrane inner side was monitored using overpressure and underpressure gauges in the filtrate tubing. After operation, the membrane rod was removed and ultrasonically cleaned with 6 M NaOH at 60 °C for at least 16 h.

### Automation of MBR operation

All state variables of the STR, e.g., temperature, pH, agitation speed, and pressure, were controlled by the bioreactor’s software (BioSCADA Lab software, Bioengineering, Wald, Switzerland). The inlet gas flow rates (CO, CO_2_, H_2_, and N_2_) were controlled individually using four thermal mass flow controllers (P-702CCV-6K0R-RAD-33-V, Bronkhorst, Reinach, Switzerland). The exhaust gas flow rate was measured using a thermal mass flow meter (F-101D-RAD-33-V, Bronkhorst, Reinach, Switzerland; N_2_-calibrated). The measured gas flow rates of all five devices were recorded at 30 s intervals using the interfaces FlowDDE and FLOW-BUS (Bronkhorst, Reinach, Switzerland). Exhaust gas composition (CO, CO_2_, H_2_, and N_2_) was analyzed at 12-minute intervals and recorded by a micro gas chromatograph with three independent channels (Micro GC 490, Agilent Technologies Inc., Santa Clara, CA, USA).

The gas uptake rates of CO were calculated at 30-minute intervals according to Eq. 1 based on the difference between the inlet flow rate of CO ($\:{\dot{\mathrm{V}}}_{\mathrm{CO},\; \mathrm{0}}$) and exhaust flow rates of CO ($\:{\dot{\mathrm{V}}}_{\mathrm{CO}}$), which were normalized to the molar volume $\:{\mathrm{V}}_{\mathrm{M}}$ (22.414 L mol^−1^) and the reactor volume $\:{\mathrm{V}}_{\mathrm{R}}$ of the STR.

For the calculation of the exhaust flow rates $\:{\dot{\mathrm{V}}}_{\mathrm{CO}}$, the gas-phase mole fractions $\:\:{\mathrm{y}}_{\mathrm{CO}}$were initially determined from the corresponding partial pressures measured with the micro GC. As the thermal mass flow meter for the exhaust gas was calibrated with N_2_, the flow rates $\:{\dot{\mathrm{V}}}_{\mathrm{Gas}}$ differed from the gas-specific flow rates. Therefore, a gas correction factor $\:\mathrm{G}\mathrm{C}\mathrm{F}$ was applied to estimate the volumetric flow rates of CO.

1 $$r_{{{\mathrm{CO}}}} ~ = ~\frac{{{{\dot{V}}}_{{{\mathrm{CO}},~0}} - ~{{\dot{V}}}_{{{\mathrm{CO}}}} }}{{{\mathrm{V}}_{{\mathrm{M}}} ~ \cdot ~{\mathrm{V}}_{{\mathrm{R}}} }} = ~\frac{{{{\dot{V}}}_{{{\mathrm{CO}},~0}} - \left[ {{\mathrm{GCF}}~ \cdot ~{{\dot{V}}}_{{{\mathrm{Gas}}}} \cdot ~y_{{{\mathrm{CO}}}} } \right]}}{{{\mathrm{V}}_{{\mathrm{M}}} ~ \cdot ~{\mathrm{V}}_{{\mathrm{R}}} }}$$

A cloud-based control software (unitelabs-sdk = = 0.3.0, UniteLabs GmbH, Munich, Germany) was used to control continuous MBR operation, acquire relevant continuous process data, and store them in a central database (Bromig et al. 2022). The individual mass flow rates of feed, filtrate, and bleed were measured gravimetrically (DS 20K0-1, Kern & Sohn GmbH, Balingen, Germany). The weights of the feed medium bottle and the filtrate containers were recorded at 20 s intervals (see Fig. 1a). The weights of the bleed collection container were recorded at 10 s intervals. The individual mass flow rates were estimated by linear regression of at least 120 weight measurements. In case of deviations from the set mass flow rate, a correction factor was determined by comparing actual and set weights over time, and the mass flow rates were automatically adjusted by controlling the speed of the corresponding peristaltic pump (Reglo ICC 4-channel, Ismatec, Grevenbroich, Germany). The control parameters were logged and stored in the Influx database.

The individual gravimetrical control scripts were started manually by setting the dilution rates, assuming a density of 1 kg L^−1^ for all liquids, as given in Table 1, starting with the medium feed and the filtrate withdrawal. The bleed withdrawal was initiated manually once the CO conversion in the gas phase reached 95%. The control scripts are available under https://github.com/bisdan/mbr-automated-flow-control.

### Analytical methods

Around 4 mL of cell broth was sampled manually for the determination of biomass, liquid substrate, and product concentrations during the process using an injection needle through a septum attached to a side port of the MBR.

For biomass determination, the optical density (OD_600_) of samples was measured in technical triplicate using a UV-Vis spectrometer (Genesys 10 S UV-Vis, Thermo Scientific, Neuss, Germany). The biomass concentration was calculated from OD_600_ using a correlation factor of 0.40 g L^−1^ OD^−1^ (Oppelt et al. 2026).

Samples were sterile-filtered for the quantification of D-fructose and the products (acetate, ethanol, and D-2,3-butanediol) using an HPLC (LC-2030 C, Shimadzu, Kyoto, Japan) equipped with a cation exchange column (Aminex HPX-87 H, Bio-Rad, Munich, Germany) and a refractive index detector (RID-20 A, Shimadzu, Kyoto, Japan). Measurements were performed using a mobile phase with 5 mM H_2_SO_4_ at a flow rate of 0.6 mL min^− 1^ and a column temperature of 60 °C under isocratic conditions.

During partial cell retention operations, a steady state was assumed once CDW and product concentrations remained within an estimation error of ± 10% for at least 1.4 mean hydraulic residence times of the biomass (equal to 5 mean hydraulic residence times of the medium). Thus, the space-time yield of the products $\:{\mathrm{STY}}_{\mathrm{P}\mathrm{,}\mathrm{i}}$ was calculated by multiplying the mean product concentrations $\:{\mathrm{c}}_{\mathrm{P}\mathrm{,}\mathrm{i}}$ under quasi-steady state conditions and the sum of dilution rates of filtrate ${\mathrm{D}}_{\mathrm{Filtrate}}$ and bleed $\:{\mathrm{D}}_{\mathrm{Bleed}}$(Eq. 2).

2 $$\:{\mathrm{STY}}_{\mathrm{P}\mathrm{,}\mathrm{i}}\mathrm{=}\:{\mathrm{c}}_{\mathrm{P}\mathrm{,}\mathrm{i}}\cdot{\mathrm{(D}}_{\mathrm{Filtrate}}+{\mathrm{D}}_{\mathrm{Bleed}})=\:{\mathrm{c}}_{\mathrm{P}\mathrm{,}\mathrm{i}}\cdot{\mathrm{D}}_{\mathrm{Feed}}$$

The space-time yield of the biomass $\:{\mathrm{STY}}_{\mathrm{X}}$ was calculated by multiplying the mean biomass concentrations $\:{\mathrm{c}}_{\mathrm{X}}$ under quasi-steady state conditions and the dilution rates of bleed $\:{\mathrm{\:D}}_{\mathrm{Bleed}}$ (Eq. 3).

3 $$\:{\mathrm{STY}}_{\mathrm{X}}\mathrm{=}\:{\mathrm{c}}_{\mathrm{X}}\cdot{\mathrm{D}}_{\mathrm{Bleed}}$$

The dilution rates $\:{\mathrm{D}}_{\mathrm{Feed}}$, $\:{\mathrm{D}}_{\mathrm{Filtrate}\mathrm{,}}\:$and $\:{\mathrm{D}}_{\mathrm{Bleed}}$ are the quotients of the corresponding flow rates and the reactor volume $\:{\mathrm{V}}_{\mathrm{Reactor}}$ (Eqs. 4–6).

4 $$\:{\mathrm{D}}_{\mathrm{Feed}}=\frac{{\dot{\mathrm{V}}}_{\mathrm{Feed}}}{{\mathrm{V}}_{\mathrm{Reactor}}}$$

5 $$\:{\mathrm{D}}_{\mathrm{Filtrate}}=\frac{{\dot{\mathrm{V}}}_{\mathrm{Filtrate}}}{{\mathrm{V}}_{\mathrm{Reactor}}}$$

6 $$\:{\mathrm{D}}_{\mathrm{Bleed}}=\frac{{\dot{\mathrm{V}}}_{\mathrm{Bleed}}}{{\mathrm{V}}_{\mathrm{Reactor}}}$$

The alcohol-to-acetate ratio $\:{\mathrm{R}}_{\mathrm{Alcohol:Acid}}$ under quasi-steady state conditions was determined by dividing the sum of the mean ethanol concentration $\:{\mathrm{c}}_{\mathrm{Ethanol}}$ and the mean D-2,3-butanediol concentration $\:{\mathrm{c}}_{\mathrm{D-2,3-butanediol}}$ by the mean acetate concentration $\:{\mathrm{c}}_{\mathrm{Acetate}}$ (Eq. 7).

7 $$\:{\mathrm{R}}_{\mathrm{Alcohol:Acid}}=\frac{{\mathrm{c}}_{\mathrm{Ethanol}}+\:{\mathrm{c}}_{\mathrm{D-2,3-butanediol}}}{{\mathrm{c}}_{\mathrm{Acetate}}}$$

The carbon balance was checked for each continuous fermentation process. The carbon recovery was calculated by summing the total molar amounts of carbon produced in biomass, acetate, ethanol, D-2,3-butanediol, and CO_2_, and dividing by the total amounts of molar carbon consumed as CO and D-fructose.

## Results and discussion

To enable transmembrane pressures across a wide operating range, continuous MBR operations were conducted at 2.5 bar_abs_. Since no process performance data from *C. autoethanogenum* LAbrini at pressures > 1 bar_abs_ were available, preliminary autotrophic batch processes were operated with varying pressures ranging from 1.0 to 2.5 bar_abs_. No clear differences in growth and product formation across the individual batch processes were observed (Figure S1).

### MBR operation with*C. autoethanogenum*LAbrini (D_Feed_= 1.4 d^−1^, autotrophic batch phase)

**Fig. 2:**
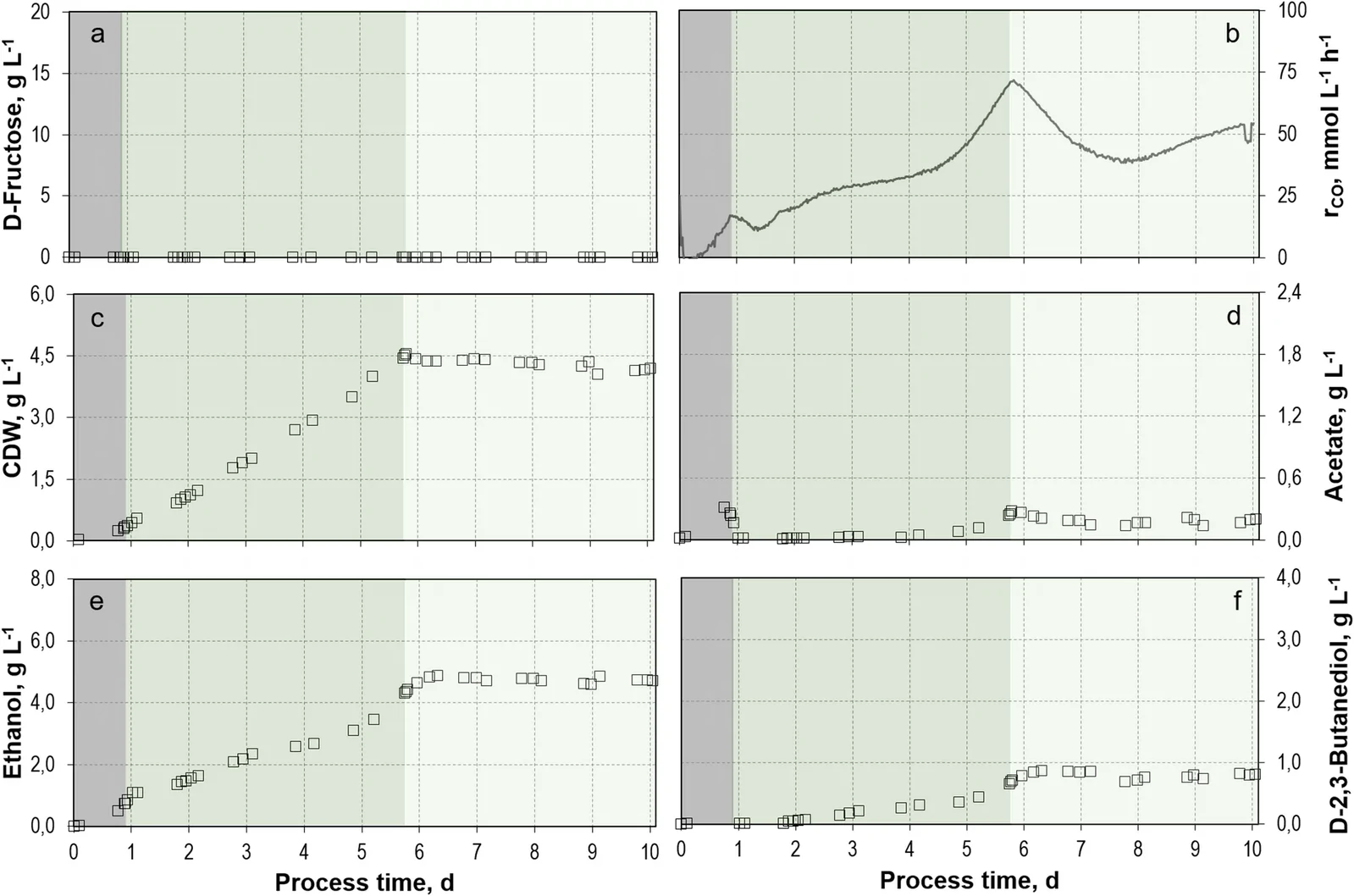
Syngas fermentation with *C. autoethanogenum* LAbrini in a continuously operated submerged membrane bioreactor at a dilution rate D_Feed_ of 1.4 d^− 1^ (*τ* = 0.71 d) with continuous gassing at 2.5 bar_abs_ (0.975 bar_abs_ N_2_, 0.75 bar_abs_ CO, 0.55 bar_abs_ H_2_, and 0.225 bar_abs_ CO_2_). The process was started with an autotrophic batch phase (dark grey), followed by a continuous phase with total cell retention (medium grey; D_Feed_ = 1.4 d^− 1^, D_Filtrate_ = 1.4 d^− 1^) and a second continuous phase with partial cell retention (light grey; D_Feed_ = 1.4 d^− 1^, D_Filtrate_ = 1.0 d^− 1^, D_Bleed_ = 0.4 d^− 1^). **a** D-Fructose concentration; **b** Carbon monoxide uptake rate; **c** Cell dry weight concentration ; **d** Acetate concentration; **e** Ethanol concentration; **f** D-2,3-butanediol concentration. (F_gas_ = 5 NL h^− 1^, 37 °C, pH 6.0, and P V^− 1^ = 15.1 W L^− 1^)

The process performance of *C. autoethanogenum* LAbrini in the submerged MBR is shown in Fig. 2. The three succeeding process phases are indicated by the varying gray-shaded areas (initial batch phase, continuous operation with total cell retention, and continuous operation with additional bleed, see Table 1, process I). The corresponding process performance data are listed in Table 2.

By the end of the initial 22-hour batch phase, the CDW concentration reached 0.3 g L^−1^, an increase by a factor of ten from the inoculation level (Fig. 2c). Acetate and ethanol were formed; no D-2,3-butanediol was produced in the batch phase (Fig. 2d,e).

After 22 h, in the exponential growth phase, continuous operation of the MBR was started with total cell retention at a feed dilution rate of 1.4 d^−1^, corresponding to two-thirds of the maximal specific growth rate estimated in the initial batch process ($D_{{{\mathrm{Feed}}}} < \mu _{{\max ,\;{\mathrm{batch}}}}$ = 2.1 d^− 1^). CDW concentration increased linearly to 4.44 g L^−1^ until the end of the continuous MBR operation with total cell retention after 5.8 d (Fig. 2c). After an initial temporary decrease in CO uptake rate, a linear increase was observed, followed by a steeper linear rise from day five onwards (Fig. 2b). Similar to the CO uptake rate, acetate production initially declined to 0.01 g L^−1^. An increase in acetate concentration was observed solely by the end of the continuous MBR operation with total cell retention, reaching a final concentration of 0.24 g L^−1^ (Fig. 2d). In contrast, the ethanol concentration increased to 4.30 g L^−1^ at the end of the process with total cell retention (Fig. 2e). D-2,3-butanediol formation started time-delayed by approximately one day, and reached 0.66 g L^−1^ (Fig. 2f).

To avoid CO limitation in the MBR with submerged membrane, an additional cell-containing effluent (bleed) was initiated once the CO conversion approached 95% (Figure S2), corresponding to a CO uptake rate of approximately 70 mmol L^−1^ h^−1^ (Fig. 2b). The bleed dilution rate (D_Bleed_) was set to 0.4 d^−1^ to compensate exactly for the linear increase in biomass concentrations during operation with total cell retention. However, a small decrease in the CDW concentration was observed in the MBR during continuous operation, reaching 4.19 g L^−1^, until process termination after 10 d (Fig. 2c).

After initiating the bleed, the CO uptake rate temporarily declined to a minimum of 38.3 mmol L^−1^ h^−1^, before increasing to a final value of 54.5 mmol L^−1^ h^−1^ (Fig. 2b). Similar to the CDW concentration, product concentrations slightly decreased (Fig. 2d–f). The continuous MBR operation with a D_Bleed_ of 0.4 d^−1^ was terminated after 6.0 mean hydraulic residence times of the medium (D_Feed_ = 1.4 d^−1^), and 1.7 mean hydraulic residence times of the biomass. The quasi-steady state product concentrations were 0.25 g L^−1^ (± 6%) acetate, 4.83 g L^−1^ (± 1%) ethanol, and 0.86 g L^−1^ (± 6%) D-2,3-butanediol, respectively, yielding a high alcohol versus acid mass ratio of 22.76. As expected, the space-time yields of the products (STY_P, i_) were much higher in the continuous MBR with partial cell retention compared to the corresponding autotrophic batch process. The STY for acetate (0.28 g L^−1^ d^−1^) was improved 2-fold, the STY for ethanol (6.76 g L^−1^ d^−1^) 15-fold, and the STY for D-2,3-butanediol (1.20 g L^−1^ d^−1^) even 40-fold (Table 2).

### MBR operation with*C. autoethanogenum*LAbrini (D_Feed_= 1.4 d^−1^, mixotrophic batch phase)

**Fig. 3:**
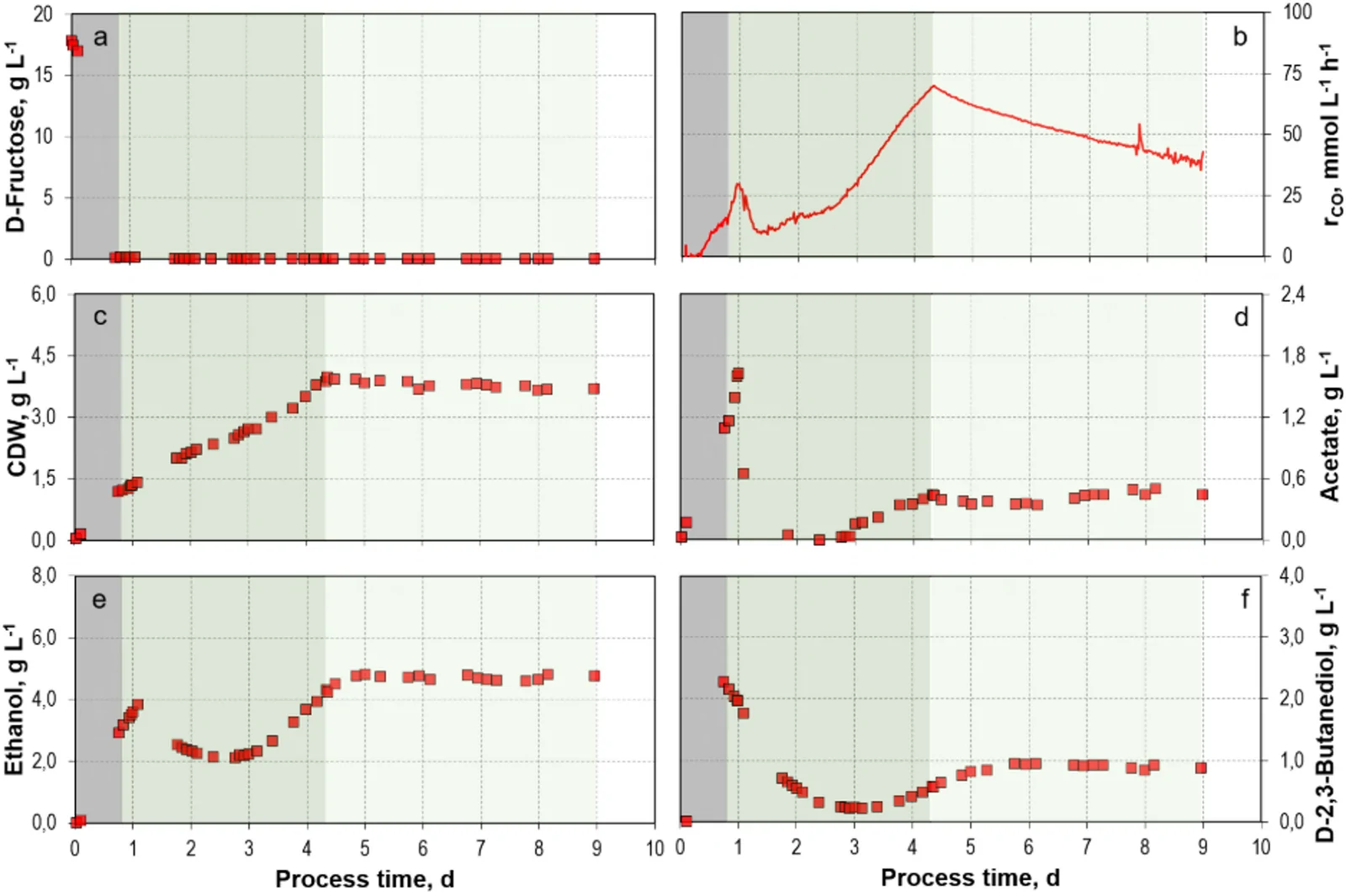
Syngas fermentation with *C. autoethanogenum* LAbrini in a continuously operated submerged membrane reactor at a dilution rate of 1.4 d^−1^ (*τ* = 0.71 d) with continuous gassing at 2.5 bar_abs_ (0.975 bar_abs_ N_2_, 0.75 bar_abs_ CO, 0.55 bar_abs_ H_2_, and 0.225 bar_abs_ CO_2_). The process was started with a mixotrophic batch phase (dark grey), followed by a continuous phase with total cell retention (medium grey; D_Feed_ = 1.4 d^−1^, D_Filtrate_ = 1.4 d^−1^) and a second continuous phase with partial cell retention (light grey; D_Feed_ = 1.4 d^− 1^, D_Filtrate_ = 1.0 d^−1^, D_Bleed_ = 0.4 d^−1^). **a** D-Fructose concentration; **b** Carbon monoxide uptake rate; **c** Cell dry weight concentration ; **d** Acetate concentration; **e** Ethanol concentration; **f** D-2,3-butanediol concentration. (F_gas_ = 5 NL h^−1^, 37 °C, pH 6.0, and P V^− 1^ = 15.1 W L^−1^)

To achieve a more efficient start-up of the MBR and, consequently, accelerate biomass accumulation for earlier 95% CO conversion, the MBR was started with a mixotrophic batch phase with an initial D-fructose concentration of 17.8 g L^−1^ the MBR was started with a mixotrophic batch phase with an initial D-fructose concentration of 17.8 g L^−1^ (Fig. 3).

During the mixotrophic batch phase, CO and D-fructose were consumed simultaneously, resulting in a biomass concentration of 1.35 g L^−1^ CDW within 22 h, exceeding the final biomass concentration after the autotrophic batch phase by more than 1 g L^−1^ (Fig. 3a–c). At the end of the batch phase, D-fructose was completely consumed, while the CO consumption rate reached 32.8 mmol L^−1^ h^−1^. High product concentrations were achieved in the mixotrophic batch phase, 1.59 g L^− 1^ acetate, 3.50 g L^−1^ ethanol, and 1.98 g L^−1^ D-2,3-butanediol, respectively (Fig. 3c–e). D-fructose was consumed 4 h earlier than in a mixotrophic batch process with *C. autoethanogenum* LAbrini at 1.0 bar_abs_ (Oppelt et al. 2026), while the final product concentrations were higher in the MBR batch phase due to operation at 2.5 bar_abs_.

As before, continuous operation with total cell retention was initiated after the batch phase at a feed dilution rate of 1.4 d^−1^. CDW concentration increased linearly within 4.5 d to 3.78 g L^−1^ (Fig. 3c). Compared to the MBR process with an autotrophic batch phase, shown in Fig. 2, the biomass concentration was thus 14% lower at the end of continuous MBR operation with total cell retention. However, the higher initial biomass concentration was associated with a more rapid increase in the CO uptake rate, reaching ~ 70 mmol L^−1^ h^−1^ at 95% CO conversion (Fig. 3b). CO uptake rate and all product concentrations initially increased upon initiating continuous operation, except for D-2,3-butanediol, before declining to a minimum after ~ 1.5 d. Acetate was fully consumed before increasing linearly to 0.44 g L^−1^ (Fig. 3d). The concentrations of ethanol and D-2,3-butanediol dropped to 2.13 g L^−1^ and 0.24 g L^−1^, respectively, before rising to 4.31 g L^−1^ and 0.58 g L^−1^ by the end of continuous operation with total cell retention (Fig. 3e,f). Continuous operation with the additional cell-containing effluent could be started 1.5 days earlier than in the MBR with an autotrophic batch phase. Here, too, the bleed dilution rate (D_Bleed_) was set to 0.4 d^−1^.

Immediately after switching on the bleed, a linear decrease of the CO uptake rate was observed to finally 31.5 mmol L^−1^ h^−1^ at the process termination after 6.4 mean hydraulic residence times of the medium (D_Feed_ = 1.4 d^−1^) and 1.8 mean hydraulic residence times of *C. autoethanogenum* LAbrini (Fig. 3c). The quasi steady state biomass concentration of 3.78 g L^−1^ decreased slightly, as before, throughout the final continuous MBR process with partial cell retention. But the observed variation of ± 2% remained well within the predefined ± 10% criterion for quasi-steady state. Also the product concentrations achieved stable quasi-steady states within the estimation error of ± 10% (Fig. 3d–f). The quasi-steady state acetate concentration of 0.41 g L^− 1^ (± 8%) was almost twice that of the MBR process with an autotrophic batch phase. The quasi-steady state alcohol concentrations were similar, with 4.72 g L^−1^ (± 2%) ethanol and 0.90 g L^−1^ (± 6%) D-2,3-butanediol. The corresponding STYs were 0.57 g L d^−1^ acetate, 6.60 g L d^−1^ ethanol, and 1.26 g L d^−1^ D-2,3-butanediol.

Summing up, a mixotrophic batch phase with an initial D-fructose concentration of 17.8 g L^− 1^ was associated with a reduction of the duration of the continuous operation with total cell retention by 1.5 days while comparable quasi-steady state alcohol concentrations and STYs were observed. However, the quasi-steady state acetate concentration and STY were higher.

### MBR operation with*C. autoethanogenum*LAbrini (D_Feed_= 2.8 d^−1^, mixotrophic batch phase)

MBRs with partial cell retention can be operated independently of the maximum specific growth rate of the microorganisms under study $(\mathrm{D}_{\mathrm{Feed}}>\mu_{\mathrm{max}})$ (Chmiel and Weuster-Botz 2018). Consequently, in a further MBR-study with a mixotrophic batch phase with D-fructose, as before, the feed dilution rate was set above the maximum specific autotrophic growth rate of *C. autoethanogenum* LAbrini ($\:{\mathrm{D}}_{\mathrm{Feed}}=\mathrm{2.8}{\mathrm{d}}^{-1}>\mathrm{2.1}{\mathrm{d}}^{-1})$.

**Fig. 4:**
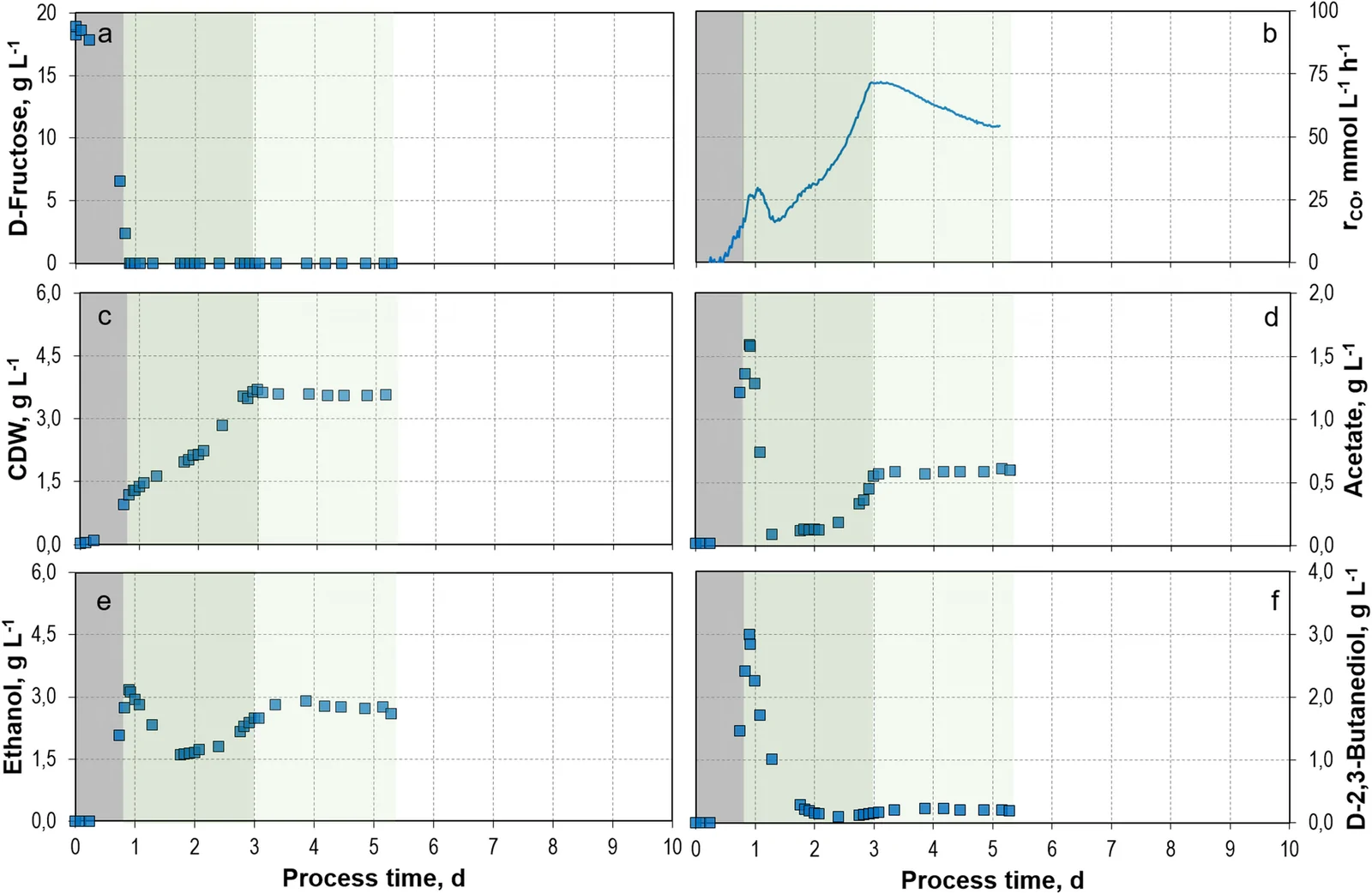
Syngas fermentation with *C. autoethanogenum* LAbrini in a continuously operated submerged membrane reactor, at a dilution rate of 2.8 d^−1^ (*τ* = 0.36 d) with continuous gassing at 2.5 bar_abs_ (0.975 bar_abs_ N_2_, 0.75 bar_abs_ CO, 0.55 bar_abs_ H_2_, and 0.225 bar_abs_ CO_2_). The process was started with a mixotrophic batch phase (dark grey), followed by a continuous phase with total cell retention (medium grey; D_Feed_ = 2.8 d^−1^, D_Filtrate_ = 2.8 d^−1^) and a second continuous phase with partial cell retention (light grey; D_Feed_ = 2.8 d^−1^, D_Filtrate_ = 2.0 d^−1^, D_Bleed_ = 0.8 d^−1^). **a** D-Fructose concentration; **b** Carbon monoxide uptake rate; **c** Cell dry weight concentration ; **d** Acetate concentration; **e** Ethanol concentration; **f** D-2,3-butanediol concentration. (F_gas_ = 5 NL h^−1^, 37 °C, pH 6.0, and P V^−1^ = 15.1 W L^−1^)

The mixotrophic batch phase in the MBR (Fig. 4) showed high reproducibility with the batch process shown before (Fig. 3). Continuous operation with total cell retention was initiated after a 22-hour batch phase, as before. Like in the MBR with a mixotrophic batch phase at D_Feed_ of 1.4 d^−1^, CDW concentrations increased linearly (Fig. 4c). Similarly, CO consumption and product formation decreased before recovering (Fig. 4b, d–f). The increased feed dilution rate D_Feed_ of 2.8 d^− 1^ was associated with more rapid process performance dynamics until a CO uptake rate of ~ 70 mmol L^−1^ h^−1^ at 95% CO conversion was reached, compared to D_Feed_ of 1.4 d^−1^. This may be attributed to the increased nutrient supply resulting from the higher feed rate. However, at the end of total cell retention phase, the CDW concentration was slightly reduced (3.64 g L^−1^ versus 3.78 g L^−1^) the acetate concentration was the same (0.45 g L^−1^), the ethanol concentration was increased (2.38 g L^−1^ instead of 2.13 g L^−1^), and the D-2,3-butanediol was reduced (0.15 g L^−1^ versus 0.24 g L^−1^), respectively, compared to the feed dilution rate applied before.

Already after 2.1 days with total cell retention, 1.25 days faster than before, the bleed was switched on with D_Bleed_ = 0.8 d^−1^. CDW and product concentrations remained stable, without an initial decrease (Fig. 4b, d–f). The CO uptake rate decreased continuously after bleed initiation to 54.3 mmol L^−1^ h^−1^ at process termination after 6.4 mean hydraulic residence times of the medium and 1.8 mean hydraulic residence times of the biomass (Fig. 4c). As in the two MBR processes shown before, during partial cell retention, a quasi-steady state was observed. While the quasi-steady state concentrations of 2.76 g L^−1^ ethanol (± 3%) and 0.21 g L^−1^ D-2,3-butanediol (± 5%) were lower than before, the acetate concentration of 0.59 g L^−1^ (± 2%) was higher (Fig. 4d–f).

**Fig. 5:**
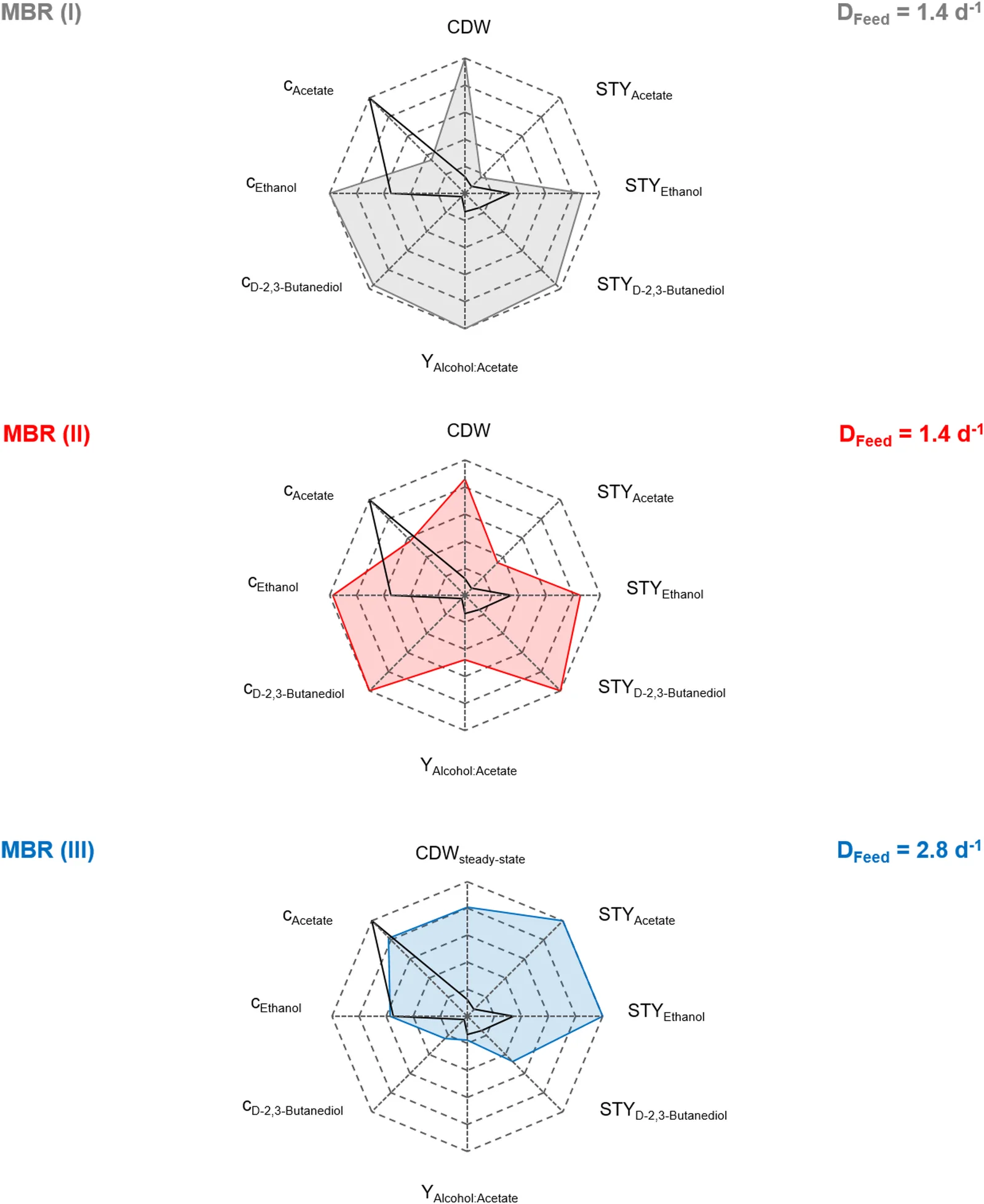
Quasi-steady state concentrations, space-time yields, and alcohol-to-acetate ratio of the three continuously operated submerged membrane reactors with continuous gassing at 2.5 bar_abs_ using *C. autoethanogenum* LAbrini compared to the final concentrations of an autotrophic batch process at 2.5 bar_abs_ (black lines). The relative process performance data are normalized to the maximum observed in the three continuous MBR processes or the reference batch process (see Table 2). (0.975 bar_abs_ N_2_, 0.75 bar_abs_ CO, 0.55 bar_abs_ H_2_, and 0.225 bar_abs_ CO_2_). (F_gas_ = 5 NL h^− 1^, 37 °C, pH 6.0, and P V^−1^ = 15.1 W L^−1^)

The autotrophic process performance of *C. autoethanogenum* LAbrini in the three continuous submerged MBR processes with partial cell retention is summarized in Fig. 5; Table 2, based on their quasi-steady state data. The submerged MBR processes with partial cell retention showed improved process performance compared to the autotrophic batch process at 2.5 bar_abs_ (Figure S1), except for the final acetate concentration, which was 0.54 g L^− 1^ in the autotrophic batch process.

**Table 2:** Process performance data in the quasi-steady state of the continuously operated submerged membrane reactors with *C. autoethanogenum* LAbrini and batch process performance data of an autotrophic batch process in a STR with continuous gassing as reference (F_gas_ = 5 NL h^−1^, 37 °C, 2.5 bar_abs_, pH 6.0, and P V^−1^ = 15.1 W L^−1^)

*C. autoethanogenum* LAbrini	Batch process	MBR		
		(I)	(II)	(III)
Batch phase	Autotroph	Autotroph	Mixotroph	Mixotroph
c_x,0_, g L^− 1^	0.03	0.03	0.03	0.03
*τ*_Medium_, d	–	0.71	0.71	0.36
*τ*_Biomass_, d	–	2.50	2.50	1.25
Process time, d	5.89	10.04	8.96	**5.28**
CDW, g L^− 1^	0.54*	**4.40** (± 3%)**	3.78 (± 2%)**	3.57(± 1%)**
Acetate, g L^− 1^	0.72*	**0.25** (± 6%)**	0.41 (± 8%)**	0.59 (± 2%)**
Ethanol, g L^− 1^	2.63*	**4.83** (± 1%)**	4.72 (± 2%)**	2.76 (± 3%)**
D-2,3-Butanediol, g L^− 1^	0.19*	0.86 (± 1%)**	**0.90** (± 6%)**	0.21 (± 5%)**
STY_CDW_, g L^− 1^ d^− 1^	–	1.76	1.51	**2.86**
STY_Acetate_, g L^− 1^ d^− 1^	0.12***	**0.28**	0.57	1.65
STY_Ethanol_, g L^− 1^ d^− 1^	0.44***	6.76	6.60	**7.73**
STY_D−2,3−Butanediol_, g L^− 1^ d^− 1^	0.03***	1.20	**1.26**	0.60
Ratio_Alcohol: Acid_, g g^− 1^	3.91	**22.76**	13.69	5.04
CO_max_ conversion, %	6.81	88.90	95.90	96.06
CO_max_ consumption, mmol L^− 1^ h^− 1^	5.50	71.78	69.93	71.76
C-balance (recovery), %	98	98	91	97

*Final CDW and product concentrations

**Values are presented as mean concentration ± coefficient of variation calculated from CDW and product concentrations within the quasi-steady state

***Space-time yields were calculated based on final CDW and product concentrations

Overall, each continuous process configuration showed distinct advantages. The MBR process with an autotrophic batch phase (D_Feed_ = 1.4 d^−1^) produced the highest ethanol concentration at quasi-steady state (4.83 g L^−1^) and the lowest acetate formation. This resulted in the superior alcohol-to-acetate ratio, albeit at the expense of an extended process duration during total cell retention to achieve 95% CO conversion. Implementing a mixotrophic batch phase with D-fructose (D_Feed_ = 1.4 d^−1^) exhibited the highest quasi-steady state concentration of 0.90 g L^−1^ D-2,3-butanediol (± 6%) and the highest volumetric D-2,3-butanediol productivity of 1.26 g L d^−1^. The MBR process with a mixotrophic batch phase at a dilution rate of 2.8 d^− 1^ had the shortest process time until switching to partial cell retention and the highest volumetric quasi-steady state ethanol productivity at 7.73 g L^−1^. Though the volumetric productivity of acetate also increased in the quasi-steady state, the acetate concentration remained low at 0.59 g L^− 1^ (± 2%). No additional fermentation products beyond acetate, ethanol, and D-2,3-butanediol were analyzed. However, *meso*-2,3-butanediol, which had been detected in our previous studies applying different cultivation conditions (Oppelt et al. 2024, Oppelt et al. 2026), was also specifically analyzed. But it was not detected in any of the experiments presented here. The carbon balances were not fully closed because organic carbon supplied by yeast extract and L-cysteine was not included in the calculations, as their conversion was not measured. Therefore, the calculated carbon recovery is based on a partial carbon balance. This disregarding likely contributed to the carbon recoveries below 100% (91–98%) observed in the MBR processes.

Increasing the dilution rate from 0.5 d^−1^ to 1.0 d^−1^ and 2.0 d^−1^ with *C. autoethanogenum* LAbrini in a continuous STR with continuous gassing resulted in increasing ethanol and D-2,3-butanediol production in the steady states, while acetate concentrations remained stable (Ingelman et al. 2025). This is in contrast to our observations in the MBR with partial cell retention, where reduced alcohol concentrations and higher acetate concentrations were measured at the higher dilution rate. However, acetate concentrations were ~ 5 g L^−1^ (Ingelman et al. 2025) in the chemostat, which is about one order of magnitude higher than in our study. The medium was without yeast extract during continuous operation in the STR, which may be one reason for the higher acetate concentrations. Furthermore, Elisiário et al. (Elisiário et al. 2023) suggested that ethanol production with *C. autoethanogenum* JA1-1 depends on the combined effects of CO mass transfer, dilution rate, pH, and undissociated acetic acid concentration rather than on dilution rate alone. Therefore, the differences observed in the present study may also reflect changes in CO availability and intracellular carbon fluxes associated with the different operating conditions.

No uptake of CO_2_ and H_2_ was observed at any time by *C. autoethanogenum* LAbrini during the MBR processes (Figure S3). This was desirable, as H₂ and CO₂ utilization would have promoted acetate formation under physiological conditions (Mock et al. 2015), potentially altering intracellular metabolic fluxes and consequently the product spectrum. After switching on the bleed for partial cell retention in the MBR processes, the CO uptake rate and, thus, CO conversion (Figure S2) always decreased. Keeping CO conversion as high as possible throughout the continuous MBR operation with partial cell retention would therefore need a sophisticated control strategy, e.g., with 95% CO conversion as a set-point. As CO conversion decreases, the bleed can be reduced while the filtrate flow is increased accordingly, and vice versa.

A constant reactor working volume was maintained throughout all MBR processes via the automated control of the flow rates of feed, filtrate, and bleed. The submerged membrane demonstrated stable, long-term performance, with no observable transmembrane pressure changes or flux decline. The specific submerged membrane area was 70.6 cm^2^ L^− 1^, which is an order of magnitude lower than in other submerged membrane bioreactor applications (Carstensen et al. 2012). The long-term microfiltration membrane stability indicates stable filtration performance of the hydrophilic sintered metal membrane surface. Characterization of the submerged microfiltration membrane by, e.g., scanning electron microscopy (SEM) at different stages of the process could provide more detailed insights into the temporal evolution of membrane surface properties. The arrangement of the submerged microfiltration membrane rod near the stirrers and the operation of the MBR at high power input generated high shear forces near the membrane surface, which may have reduced the likelihood of biofilm formation. In addition, the relatively low permeate flow rates of 41.65–116.49 mL h^− 1^ and the typically low biomass concentrations in gas fermentations during MBR operation (3.57–4.4 g L^−1^, Table 2) may have supported stable long-term operation of the microfiltration membrane.

## Conclusions

Partial cell retention with a continuously operated MBR enabled higher biomass concentrations (factor 6.6–8.1) and, thus, increased space-time yields of acetate, ethanol, and D-2,3-butanediol during syngas conversion with *C. autoethanogenum* LAbrini compared to the corresponding batch process. However, the alcohol concentrations were improved solely in the MBR at a medium dilution rate (D_Feed_) below the maximum growth rate of *C. autoethanogenum* LAbrini. As a consequence, the operating point of an MBR in gas fermentation should be carefully chosen. Due to the considerable operational effort and reactor occupancy required for these long-term continuous cultivations, repeating the experiments was not feasible within the scope of this study. Nevertheless, process phases conducted under identical conditions (e.g., the mixotrophic batch phase) showed very good agreement across experiments. Furthermore, the observed trends were consistent across the cultivations, supporting the validity of the presented results despite the absence of biological replicates. However, in the future, modeling and simulation will be necessary to identify optimum MBR operation and to keep the operational load low. Furthermore, the measurement of dissolved gas concentrations in future experiments will enable a more detailed assessment of potential gas-transfer limitations, and a conventional continuous STR study may be performed in the future to compare process performances with and without cell retention.

Applying an initial mixotrophic batch phase with fructose as the heterotrophic carbon source was associated with shorter process times until the initiation of continuous MBR operation with partial cell retention. However, the quasi-steady states were different. Less biomass and more acetate were produced even 8 days after the mixotrophic batch phase. This demonstrates the long-term effects of initial reaction conditions in gas fermentations, illustrated here using *C. autoethanogenum* LAbrini as an example.

The submerged microfiltration membrane rod made of steam-sterilizable, sintered metal is a very promising approach for MBR applications in syngas fermentations showing long-term operation stability at low biomass concentrations (< 5 g L^−1^) and low specific permeate flow rates (< 1.65 mL cm^−2^ h^−1^). Due to the low specific membrane area of ~ 70 cm² L⁻¹ demonstrated here, submerged MBRs show potential for scalability in syngas fermentation, addressing a major limitation of this type of bioreactor in microbial biotechnology.

## Supplementary Information

Below is the link to the electronic supplementary material.

Supplementary Material 1.

## Data Availability

All data generated or analyzed during this study are included in the published article or in the supplementary information.
