# Supplementary material for: Continuous autotrophic production of alcohols with Clostridium autoethanogenum LAbrini in a submerged membrane bioreactor
Source: Bioresour Bioprocess. 2026 Sep 26;13(1):143. doi: 10.1186/s40643-026-01128-y (PMC13615957; doi:10.1186/s40643-026-01128-y)
Supplement: Supplementary file 1 [file 40643_2026_1128_MOESM1_ESM.docx]

**Supporting information**

**Table S1:** Composition of the cultivation medium used for precultures and membrane bioreactor processes.

| Component | Concentration in Stock solution, g L^‑1^ |
| --- | --- |
| Mineral solution | **33.3x** |
| Ammonium chloride | 100 |
| Natrium chloride | 80 |
| Potassium chloride | 10 |
| Potassium dihydrogen phosphate | 10 |
| Magnesium sulfate | 20 |
| Calcium chloride | 4 |
| Vitamin solution | **100x** |
| Pyridoxine | 0.01 |
| Thiamine | 0.005 |
| Riboflavine | 0.005 |
| Calcium pantothenate | 0.005 |
| Liponic acid | 0.005 |
| Para amino benzoic acid | 0.005 |
| Nicotinic acid | 0.005 |
| Vitamin B12 | 0.005 |
| D-biotine | 0.002 |
| Folic acid | 0.002 |
| 2-mercaptoethanesulfonic acid | 0.02 |
| Trace element solution | **100x** |
| Nitrilotriacetic acid | 2.00 |
| Mangan sulfate | 1.00 |
| Ammonium iron sulfate | 0.80 |
| Cobalt chloride | 0.20 |
| Zinc sulfate | 0.20 |
| Copper chloride | 0.02 |
| Nickel chloride | 0.02 |
| Sodium molybdate | 0.02 |
| Sodium selenate | 0.02 |
| Sodium wolframate | 0.02 |
|  | **Medium concentration, g L^‑1^** |
| Yeast extract | 1.0 |
| Cysteine hydrochloride ^a)^ | 0.4 |
| Morpholino ethane sulfonic acid^b)^ | 15.0 |

a) not used for preculture in anaerobic shaken bottles

b) only used for preculture in anaerobic shaken bottles

**Figure S1.** Autotrophic batch processes of *C. autoethanogenum* LAbrini at varying system pressure ( 1.0 bar_abs_,  1.5 bar_abs_,  2.0 bar_abs_ and  2.5 bar_abs_) in stirred-tank bioreactors with continuous gassing (artificial gas mixture of N_2_, CO, CO_2_, and H_2_ in a ratio of 39:30:22:9). (F_gas_ = 5 NL h^-1^, 37 °C, pH 6.0, and P V^‑1^ = 15.1W L^‑1^). (a) System pressure; (b) Carbon monoxide uptake rate; (c) Cell dry weight concentration (CDW); (d) Acetate concentration; (e) Ethanol concentration; (f) D-2,3-butanediol concentration. The error bars indicate the minimum and maximum values of two individual autotrophic batch processes.

**Table S2.** Process performance data of autotrophic batch processes of *C. autoethanogenum* LAbrini at varying system pressure.

| **System pressure, bar_abs_** | **1.0**  **min/max** | **1.5** | **2.0** | **2.5** |
| --- | --- | --- | --- | --- |
| µ_max_, h^-1^ | 0.08-0.09 | 0.10 | 0.12 | 0.09 |
| CDW, g L^-1^ | 0.63-0.65 | 0.52 | 0.50 | 0.54 |
| c_Acetate,final_, g L^-1^ | 0.75-1.20 | 0.52 | 0.53 | 0.72 |
| c_Ethanol,final_, g L^-1^ | 2.33-2.74 | 2.14 | 2.17 | 2.63 |
| c_D-2,3-Butanediol,final_, g L^-1^ | 0.31-0.37 | 0.17 | 0.17 | 0.19 |
| Ratio_Alcohol,final:Acetate,final_, g g^‑1^ | 2.20-4.14 | 4.45 | 4.39 | 3.91 |
| CO consumption_max_, mmol L h^-1^ | 6.45-8.70 | 5.64 | 4.60 | 5.50 |
| C-balance (recovery), % | 96.44-99.82 | 101.16 | 96.66 | 93.09 |

**Figure S2.** CO availability of continuously operated submerged membrane reactors ( MBR (I),  MBR (II),  MBR (III)) for syngas fermentation with *C. autoethanogenum* LAbrini with continuous gassing at 2.5 bar_abs_ (0.975 bar_abs_ N_2_, 0.75 bar_abs_ CO, 0.55 bar_abs_ H_2_, and 0.225 bar_abs_ CO_2_). (F_gas_ = 5 NL h^-1^, 37 °C, pH 6.0, and P V^‑1^ = 15.1W L^‑1^).

**Figure S3.** Gas rate of continuously operated submerged membrane reactors ( MBR (I),  MBR (II),  MBR (III)) for syngas fermentation with *C. autoethanogenum* LAbrini with continuous gassing at 2.5 bar_abs_ (0.975 bar_abs_ N_2_, 0.75 bar_abs_ CO, 0.55 bar_abs_ H_2_, and 0.225 bar_abs_ CO_2_). Solid colored lines present CO uptake rates, solid grey lines indicate CO_2_ production rates, and dashed grey lines represent H_2_ rates. Vertical red lines mark five mean hydraulic retention times *τ*. (F_gas_ = 5 NL h^-1^, 37 °C, pH 6.0, and P V^‑1^ = 15.1W L^‑1^).
